# Androgen deficiency is associated with a better prognosis in glioblastoma

**DOI:** 10.1186/s40001-024-01648-3

**Published:** 2024-01-17

**Authors:** Helga Fariña-Jerónimo, Rita Martín-Ramírez, Rebeca González-Fernández, Lilian Medina, Antonia de Vera, Pablo Martín-Vasallo, Julio Plata-Bello

**Affiliations:** 1 Neurosurgery Department, Hospital Universitario de Canarias, Calle Ofra s/n La Cuesta, CP 38320 La Laguna, S/C de Tenerife Spain; 2 Molecular Biology Department, University of La Laguna, La Laguna, Spain; 3 Biochemistry Laboratory, Hospital Universitario de Canarias, La Laguna, Spain

**Keywords:** Glioblastoma, Testosterone, Androgen receptor, Prognosis

## Abstract

**Background:**

The androgen receptor (AR) has been demonstrated to play a role in the pathogenesis of glioblastoma; however, the implications of circulating testosterone levels in the biology of glioblastoma remain unknown.

**Aim:**

This study aimed to analyze the association between circulating testosterone levels and the prognosis of patients with glioblastoma.

**Methods:**

Forty patients with primary glioblastoma were included in the study. The main prognostic endpoint was progression-free survival (PFS). Circulating testosterone levels were used to determine the state of androgen deficiency (AD). AR expression was analyzed by reverse-transcriptase polymerase chain reaction, Western blot, and immunofluorescence. Survival analysis was performed using the log-rank test and univariate and multivariate Cox regression analysis.

**Results:**

Most of the patients showed AR expression, and it was mainly located in the cytoplasm, as well as in the nucleus of tumor cells. Patients with AD presented a better PFS than those patients with normal levels (252.0 vs. 135.0 days; *p* = 0.041). Furthermore, normal androgenic status was an independent risk factor for progression in a multivariate regression model (hazard ratio = 6.346; *p* = 0.004).

**Conclusion:**

Circulating testosterone levels are associated with the prognosis of glioblastoma because patients with AD show a better prognosis than those with normal androgenic status.

## Introduction

Glioblastoma is the most common primary brain tumor in adults, with an incidence of approximately four cases/100,000 inhabitants/year [1–3], although it can be even higher in elderly patients, with an overall incidence rate of 13.16 cases per 100,000 inhabitants [4] Glioblastoma is associated with a very bad prognosis (mean overall survival (OS) of 12–15 months) [1, 2, 5], and despite the use of standard treatment (surgery and radiochemotherapy [Stupp Scheme]), only 5% of the patients survive for 5 years after diagnosis [2]. In this regard, a better understanding of the biology of glioblastoma is essential to the identification of new therapeutic targets.

Sex hormone receptors are one of those potential targets. The role of sex hormone receptors in glioblastoma has already been analyzed [6]. Progesterone, estrogen, and androgen receptors are expressed in glioblastoma tissues, and they have been demonstrated to play a role in glioblastoma pathogenesis, modulating cell growth, migration, and invasion [7–12]. In vitro studies have revealed that physiological doses of progesterone may be linked to increased aggressiveness in glioblastoma cells, whereas higher doses of progesterone appear to exhibit an anti-tumoral effect. Additionally, the activation of the estrogen receptor in glioblastoma cells follows a dose-dependent pattern. A special focus on the androgen receptor (AR) and its pathogenic role in glioblastoma has been provided in the last decade. The AR is a type of nuclear receptor that is mainly activated by the binding of androgens, mainly testosterone and dihydrotestosterone. The inactivated AR is mainly located in the cytoplasm. The union of an androgen molecule produces a conformational change in the receptor and a dissociation from heat-shock proteins. The activated AR translocates to the nucleus, dimerizes, and binds to specific hormone response elements to regulate the expression of certain genes (called androgen-responsive genes [ARGs]) [13, 14].

A higher AR expression in glioblastoma biopsies than in the normal brain has been reported [12, 15–17], and AR expression has been associated with the histological grade of glial tumors; a high AR expression is found whenever the tumoral grade increases [18]. Regarding the role of the AR in glioblastoma, its activation has been associated with increased proliferation of glioblastoma cells and increases in their migration and invasiveness capacity [17, 19, 20]. Furthermore, in vitro and in vivo studies have shown that silencing the AR gene or its pharmacological blockade led to tumoral cell death [15, 16, 18, 21]. A study recently demonstrated that enzalutamide (an anti-androgen) not only inhibits the proliferation of glioblastoma cells both in vitro and in vivo but also targeted glioma stem cells (GSCs) [16]. Interestingly, a cross-talk between the AR and epidermal growth factor receptor (EGFR) pathways has been described in glioblastoma cells [11] and AR activation in vitro regulates transcription programs related to radiation-induced DNA damage repair [22]. Finally, a recent in silico study demonstrated a worse prognosis in patients with higher AR activity [23].

Therefore, AR activity plays a role in the biology of glioblastoma. However, the implications of circulating testosterone levels in AR activity in glioblastoma have not been analyzed yet. Testosterone levels were reported to be higher in patients with gliomas than in patients with other neurosurgical diseases, such as benign tumors or craniocerebral trauma [18]. Additionally, the difference in the incidence rates of glioblastoma reported by sex (3:2, men:women) reinforced the hypothesis that a more androgenic environment facilitates glioblastoma development [2], but this must be confirmed.

With the above, this study aimed to analyze the effect of circulating testosterone levels on the prognosis of patients with glioblastoma and AR activity and to identify any clinical, radiological, or molecular difference between patients with normal or abnormal testosterone levels. The identification of a possible association between the androgenic status and the prognosis in glioblastoma could facilitate the use of the AR as a plausible therapeutic target.

## Methods

### Study design

A prospective observational study was conducted.

### Patients

Forty consecutive patients with primary glioblastoma (IDH1 and 2 wild-type), diagnosed and treated in our center, were included in the study (mean age, 62.7 years; 16 women). The World Health Organization (WHO) central nervous system (CNS) from 2016 was used for tumor classification by two independent pathologists. The first patient was enrolled in May 2019, and the last patient in September 2021. After the surgery, all patients were managed following the standard of care, with chemoradiotherapy (temozolomide) at standard doses. The sample size was estimated in 27 patients with a 95% confidence level (CI) and 3% precision to determine a minimum hazard ratio of 3.0 (HR = 3.0) of an interest variable. Molecular data were only available for 28 patients (although ultimately only 25 were included in the analysis due to a lack of some clinical data). The methylation status of the MGMT promoter and expression of Ki-67 were extracted from pathological reports for every patient included in the study.

All participants provide informed consent, and the study was approved by the local ethics committee.

### Hormonal blood level measurement

Blood probes were obtained from 8:00 to 9:00 AM before the surgery (range, 1–6 days). No steroids were administered before blood probe acquisition. The levels of luteinizing hormone (LH), follicle-stimulating hormone (FSH), sex hormone binding globulin (SHBG), and total testosterone were measured using the IMMULITE 2000 analyzer. SHBG, FSH, and LH were measured in solid-phase, two-site chemiluminescent immunometric assays. Total testosterone was measured in a solid-phase, competitive chemiluminescent enzyme immunoassay. Free testosterone was analyzed on MAGLUMI® analyzer. Free testosterone levels were measured in competitive chemiluminescence immunoassays. The free androgen index (FAI) was calculated for each patient following the formula proposed by [24]:

$$\# {\text{ FAI }} = \, ({\text{total testosterone}}/{\text{ SHBG}}) \times {1}00.$$

Hormonal levels were available for 35 patients. Androgen deficiency (AD) and its origin (primary or secondary) were evaluated following the recommendations of current clinical practice guidelines [25, 26]. Patients with and without AD were compared.

### Tissue sample handling

Once surgical probes were obtained, two tumor pieces of 1–3 mm^2^ were stored at − 80 °C at the Biobank of our center. One piece was submerged in TRIzol™ (Thermo Fisher Scientific, Waltham, MA, USA) for RNA extraction, and the other piece was submerged in formaldehyde and, posteriorly, embedded in paraffin for immunohistochemical analyses.

### Quantitative reverse-transcription polymerase chain reaction (qRT-PCR)

Total RNA was isolated using TRizol reagent following the manufacturer’s instructions. Reverse-transcription reactions were conducted using iScript cDNA Synthesis kit (Bio-Rad Laboratories, Hercules, CA, USA), following the manufacturer’s instructions.

PCRs were performed in a Bio-Rad CFX96 real-time PCR system (Bio-Rad Laboratories) using 2 × Sso Fast Eva Green Supermix (Bio-Rad Laboratories) and 0.4 µmol/L of each primer in 10 µL of the final volume. Specific primers for each gene of interest amplification were as follows (5’ → 3’):

AR (F: AATCCCACATCCTGCTCAAG, R: AAGTCCACGCTCACCATG)

MCEE (F: CAACCATGTAGCCATAGCAGTGC, R: TCCATGTTCAGGAAGAGGGACC)

SLC26A2 (F: CAGATACCTCTGAGGACCTACC, R: CAACATGCTCCACAAAGC)

FKPB5 (F: TAGCCTCCTCCCAAAGTCC, R: CTAATCCAGAAACTCTCATCTGC)

VEGFA (F: ACAACAAATGTGAATGCAGACC, R: ACACGCTCCAGGACTTATACC)

SLC22A3 (F: ATCGTCATTTACTTGCTATCCTGC, R: CGTCCCCTTTCCAAATACACC)

KLF4 (F: ACCTACACAAAGAGTTCCCATC, R: TGTGTTTACGGTAGTGCCTG).

All samples were analyzed in triplicate using the following thermal profile: after 30 s of initial denaturation at 95 °C, 45 cycles of PCR were performed at 95 °C for 5 s and 59 °C for 5 s. Finally, a melting curve program at 65 °C to 95 °C was conducted with a heating rate of 0.1 °C/s and read every 0.5 °C. The expression levels of the genes studied are presented as individual data points as 2ΔCT [27].

### Protein expression analysis by Western blot (WB)

Thirteen tissues samples homogenized in Laemmli sample buffer were electrophoresed on a denaturing 8% polyacrylamide gel and transferred to Immobilon™-P membranes (Millipore, Bedford, MA, USA) by electroblotting. After blocking in phosphate-buffered solution/5% bovine serum albumin (BSA) for 1 h, protein detection was performed overnight at 4°C using mouse AR antibody (441) dilution 1:250 (sc-7305, Santa Cruz Biotechnology Inc., Dallas, TX, USA) and anti-mouse Ig sheep peroxidase (A5906, Sigma–Aldrich, Merck Life Science S.L.U., Madrid, Spain) secondary antibody (1:5000). Detection was performed using Immobilon Western Chemiluminescent HRP substrate (Millipore, Merck Life Science S.L.U.), according to the manufacturer’s instructions, in a ChemiDoc XRS (Bio-Rad Laboratories). Band densities were measured using Image Lab analysis software (Bio-Rad Laboratories) relative to total proteins per lane.

### AR activity estimation: AR score calculation

To infer the AR activity, the expression of previously validated ARGs was determined [28]. Six ARGs were selected from a group of 13 that have been previously analyzed in HPr-1AR (normal prostate cell line) and LNCaP (prostate cancer cell line) cells [28] and that have been associated with a worse prognosis in glioblastoma [23]. These genes are those whose primers are listed above (i.e., MCEE, SLC26A2, FKPB5, VEGFA, SLC22A3, and KLF4). As previously described elsewhere [29], AR activity was defined by the quantification of the composite expression of this six-gene signature in each sample. As in other works [23, 30], a Z-score was computed for the expression of each gene in each sample by subtracting the pooled mean from the RT-PCR expression values and dividing the result by the pooled standard deviation. The AR putative activity (called AR score) for each sample was then computed as the sum of the Z-scores of the ARG signature. The median AR score (p50) was used to analyze differences between patients with high or low AR activity. As previously indicated in the qRT-PCR section, this measure was only available for 25 patients.

### Immunofluorescence

In this study, 5 µm thick, 10% formalin-fixed paraffin-embedded tissue sections of 16 patients were deparaffinized in xylene and hydrated in a graded series of alcohol baths. After heat-induced epitope retrieval autoclaving samples at 120 °C for 10 min in sodium citrate buffer (pH 6.0), non-specific sites were blocked with 5% BSA in Tris-buffered saline for 1 h at room temperature.

AR immunofluorescence staining was performed. Tissue sections were incubated overnight at 4 °C, simultaneously with rabbit polyclonal AR antibody dilution 1:200 (PA1-110, Thermo Fisher Scientific). After three washes, the samples were incubated for 1 h at room temperature in the dark and mixed with two secondary antibodies: fluorescein isothiocyanate (FITC)-conjugated goat polyclonal antibody against rabbit IgG (dilution 1:200; #F9887; Sigma–Aldrich, Merck Life Science S.L.U.). Finally, samples were mounted with ProLong®Diamond Anti-fade Mountant with DAPI (Molecular Probes by Life Technologies) and analyzed under Leica SP8 (Leica Microsystems, Wetzlar, Germany) confocal microscope.

Apart from the qualitative description of the immunofluorescence images, the mean intensity of the AR fluorescence in the nucleus and in the rest of the cell was measured using Fiji (https://imagej.net/software/fiji/). A mask with nuclei was firstly performed for each image; afterwards, the mean intensity of AR fluorescence was measured within the areas of this mask. An index of the relative mean intensity in the nucleus to the mean global intensity of the AR immunofluorescence was calculated for each patient (so-called AR_n/t_).

### Magnetic resonance imaging (MRI)

All the studied patients had presurgical MRI data available. The OncoHabitats platform (https://www.oncohabitats.upv.es/) was used to measure three kinds of glioblastoma-related volumes: enhancing tumor, necrosis, and edema volumes. This platform enables MRI preprocessing in combination with automated segmentation of the above-mentioned volumes based on convolutional neural networks [31]. Three patients were dropped from the study because the MRI volume assessment was affected by excessive movement during image acquisition, which compromised the quality of the study. The segmentation of the rest of the presurgical MRIs (*n* = 37) was visually inspected to confirm the absence of any bias. Apart from the segmentation, the contrast enhancement pattern (ring/peripheric vs. heterogeneous) was evaluated by two experienced neurosurgeons. Furthermore, the necrosis-to-contrast ratio was also calculated from the data provided by Oncohabitats.

### Statistical analysis

Nonparametric statistical tests were used to compare groups of patients (low vs. high AR score; AD vs. non-AD). In this regard, the Mann–Whitney U was used to analyze continuous variables (e.g., hormonal levels, AR expression, and AR score), and Fisher’s exact test or Chi-square was used to analyze discrete variables (e.g., sex, extent of resection, and methylation). Correlation analysis (Spearman’s Rho) was conducted between AR expression at RNA and protein levels.

In this study, the main prognostic endpoint was progression-free survival (PFS), defined as the time from treatment initiation until disease progression or worsening. Survival analysis was performed using Kaplan–Meier curves and the log-rank test.

Furthermore, a univariate Cox regression analysis was performed to calculate the HR of the association between AD and PFS and OS. Furthermore, these variables were included in the multivariate model, which also included other variables that have been demonstrated to be associated with prognosis in glioblastoma: age, presurgical Karnofsky performance status (KPS), extent of resection, and MGMT status. Significance for all analyses was considered when *p* < 0.05.

## Results

### AR is widely expressed in tumoral cells of glioblastoma

**Fig. 1:**
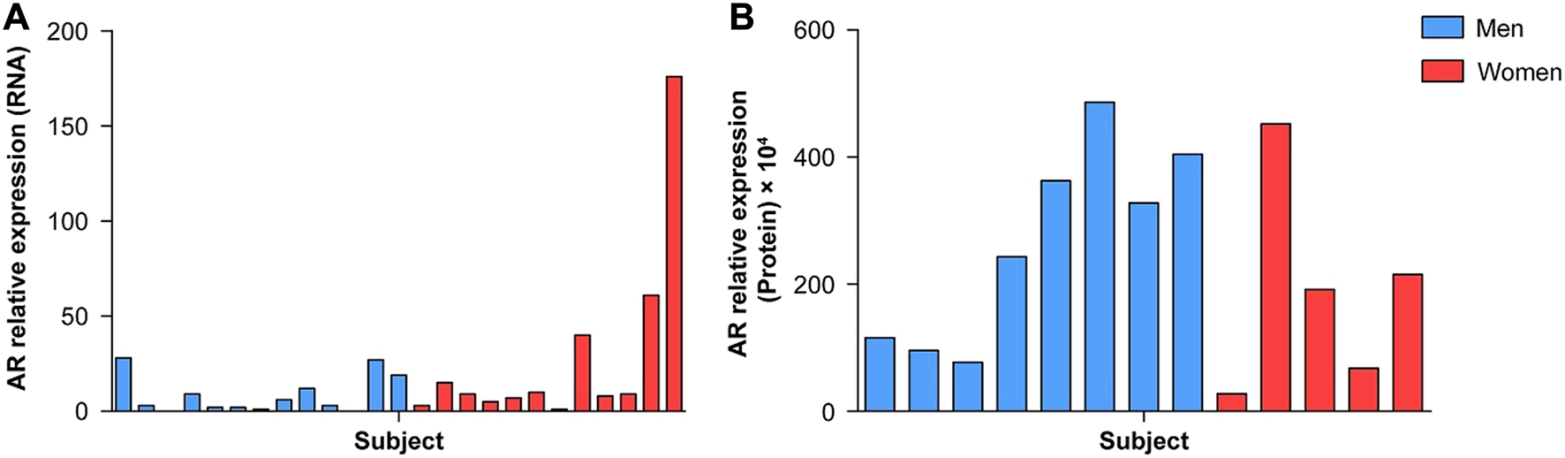
Expression of the androgen receptor (AR) in glioblastoma probes. **A** AR relative expression at the RNA level in each patient with available probes for reverse-transcription polymerase chain reaction. **B** AR relative expression at the protein level in each patient with available probes for Western blot

**Fig. 2:**
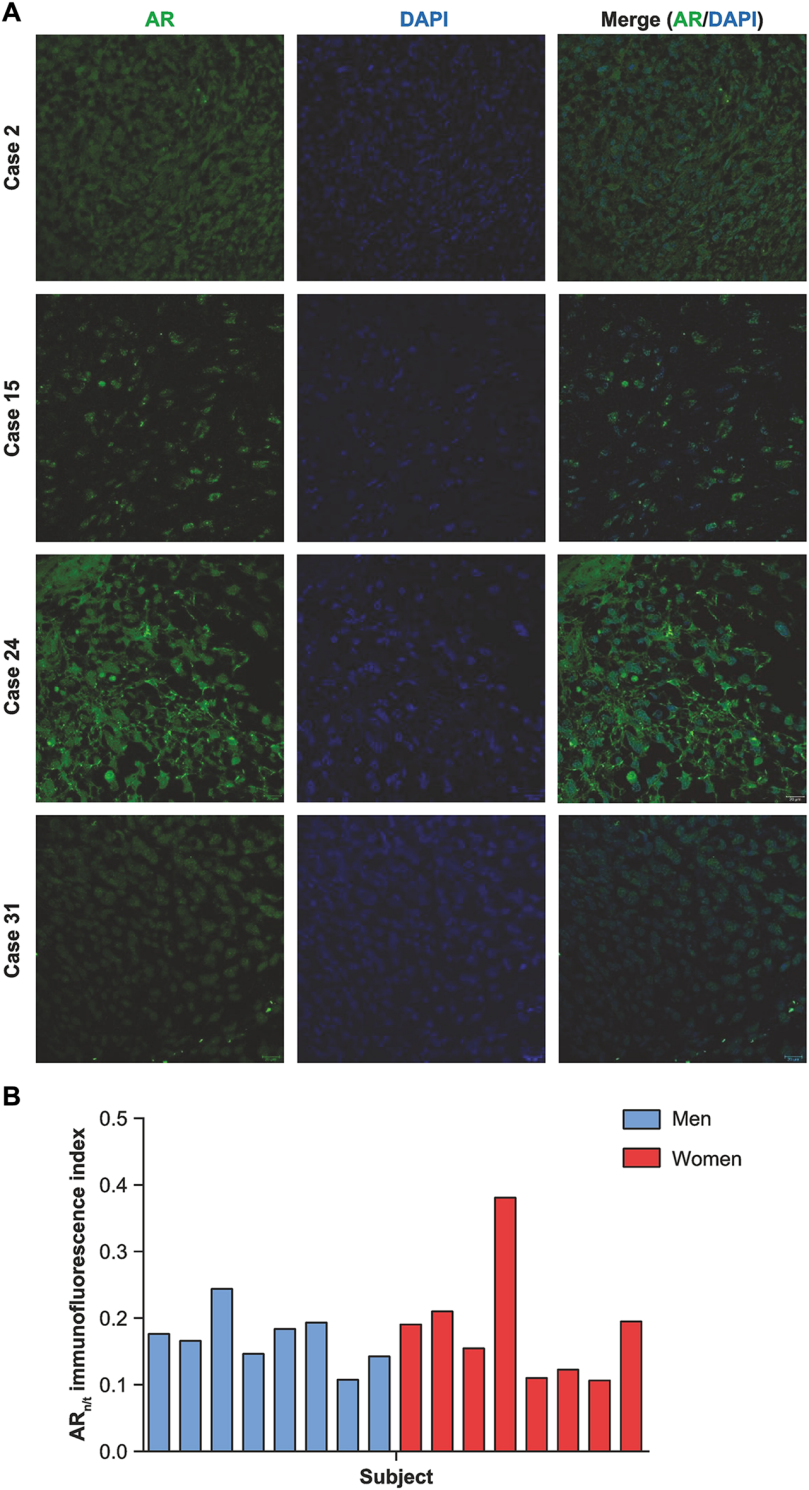
Androgen receptor (AR) immunofluorescence. **A** Examples of immunofluorescence of the AR in different glioblastoma cases. Blue, DAPI-stained nucleus. **B** Bars represent the mean ARn/t immunofluorescence index in each patient

AR expression in glioblastoma samples was assessed both at RNA (qRT-PCR) and protein levels (WB and immunofluorescence). All patients presented some levels of AR expression (Fig. 1A, B), although no correlation was identified between WB and qRT-PCR results for each patient (CC =  − 0.052; *p* = 0.849). AR immunofluorescence (*n* = 16) was mainly located in the cytoplasm of glioblastoma cells, as well as the nucleus, which showed variable intensity among patients (Fig. 2A, B). In some patients, nuclear aggregates of fluorescence were observed, and in others, a higher intensity was observed in cellular processes. The mean AR_n/t_ fluorescence index was 0.17 (SD = 0.07), and no significant difference was found between men and women (mean AR_n/t_ 0.17 vs. 0.18; *p* = 0.916) (Fig. 2B).

### The AR showed variable activity in patients with glioblastoma

**Fig. 3:**
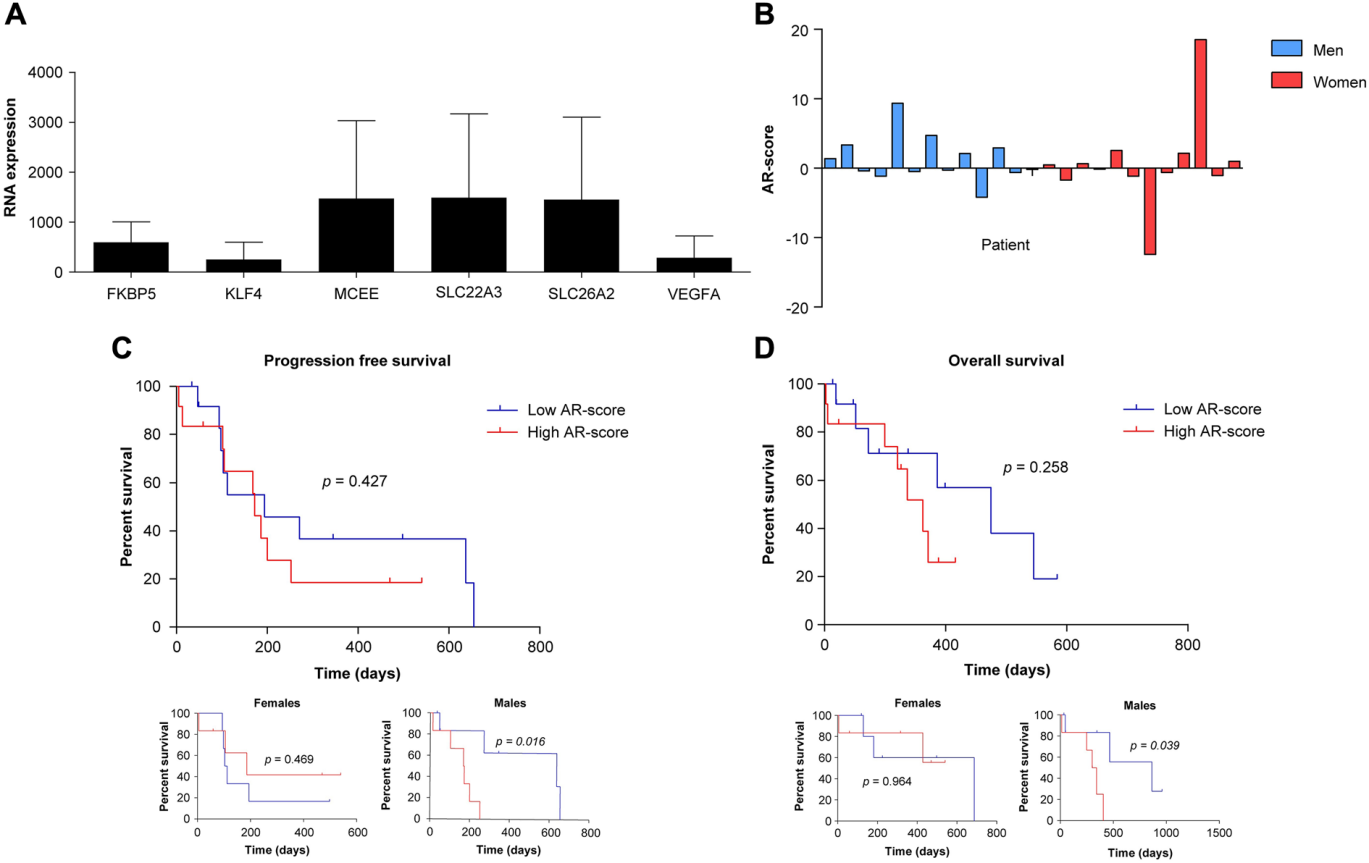
Androgen receptor (AR) activity in glioblastoma is inferred by the AR score**.** **A** Mean expression of the selected androgen-responsive genes (bars represent the statistical deviation) that were used to compute the AR score. **B** AR score in each patient with glioblastoma with available molecular data. **C** Progression-free survival analysis between patients with low and high AR scores (cutoff = p50), overall and by sex (inferior row). **D** Overall survival analysis comparing patients with low and high AR scores (cutoff = p50), overall and by sex (inferior row)

The location of the AR in the nucleus may represent an activated state of the receptor, binding to specific androgen-response elements and promoting the expression of some ARGs. Therefore, to determine AR activity in glioblastoma samples, the expression of specific ARGs was determined to calculate the so-called AR score (Fig. 3A, B). The median AR score (p50 =  − 0.15) was used to analyze differences between patients with high or low AR activity (Table 1). Patients with high AR scores presented higher percentages of Ki67 positivity (31.78 vs. 22.77) and lower necrosis-to-contrast ratio (0.26 vs. 0.86). However, these differences did not reach significance (*p* > 0.05). The only significant difference between patients with high and low AR scores was the distribution of the extent of the resection; the proportion of complete resections was higher in the low AR score group than in the high AR score group. However, no difference in survival analysis (PFS or OS) was identified for the whole group, but when analyzing the effect of the AR score in each sex, a worse PFS and OS was found in men with high AR scores (Fig. 3C, D). It should be noted that there was no follow-up loss. The mean follow-up period until progression was 195.7 (SD = 186.97) days, and the mortality rate was 40%.

**Table 1:** Comparison between patients with low and high AR-Score

	AR-Score		*p*-value
	Low (*n* = 13)	High (*n* = 12)	
Age (years)	64.31 (SD = 14.00)	66.08 (SD = 7.33)	0.744^1^
Gender (male:female)	7:6	6:6	1.0^2^
Karnofsky < 70	–	2 (16.7%)	0.125^2^
Biopsy/partial			
Resection	–	2 (16.7%)	0.023^2^
Subtotal	1 (7.7%)	5 (41.7%)	
Total	12 (92.3%)	5 (41.7%)	
Ki67	22.77 (SD = 17.64)	31.78 (SD = 20.34)	0.076^1^
MGMT methylation	6 (46.2%)	5 (41.7%)	1.0^2^
Pattern of contrast enhancement			
Peripheric	6 (46.2%)	6 (50.0%)	1.0^2^
Heterogeneous	7 (53.8%)	6 (50.0%)	
Enhancing tumor (cc)	18.03 (SD = 15.57)	22.22 (SD = 10.44)	0.424^1^
Edema (cc)	70.39 (SD = 39.92)	43.13 (SD = 23.30)	0.140^1^
Necrosis (cc)	9.38 (SD = 8.65)	6.66 (SD = 9.66)	0.324^1^
Necrosis-to-contrast ratio	0.86 (SD = 0.99)	0.26 (SD = 0.32)	0.074^1^
AR expression (WB) (Z-value)	-0.30 (SD = 1.01)	-0.49 (SD = 0.84)	0.188^1^
ARn/t fluorescence	0.15 (SD = 0.05)	0.19 (SD = 0.07)	0.278^1^
Androgen deficiency (n = 21)	5 (45.5%)	6 (60.0%)	0.670^2^
Progression-free survival (days)	194.0 [0–449.5]	172.0 [143.3–200.6]	0.427^3^
Men	637.0 [73.7–1200.3]	168.0 [84.0–252.0]	0.016^3^
Women	103.0 [86.2–119.8]	186.0 [17.0–355.0]	0.469^3^
Overall survival (days)	687.0 [246.7–1127.3]	406.0 [274.4–537.6]	0.258^3^
Men	864.0 [209.7–1518.3]	301.0 [211.7–390.3]	0.039^3^
Women	474.0^a^ [193.8–754.5]	420.0^a^ [263.2–576.2]	0.964^3^

^1^Mann–Whitney *U* test

^2^Fisher’s exact test or Chi-square

^3^Log-rank test

^a^This value represents the mean period, while the mean was cannot be computed because of the limited follow-up

### Relationship between AD and prognosis

Mean level of the free testosterone levels in participants with available hormonal data was 6.12 ng/dl (SD = 9.32). Considering the definition of AD [25, 26], most of the study patients showed abnormalities in their circulating androgen levels, according to their sex and/or age. More specifically, 72.2% (*n* = 26, 11 women) of the patients with available hormonal data presented low total testosterone levels and 51.4% (*n* = 18, 5 women) presented low free testosterone levels. All these patients, except one, presented low FAI levels. Patients with low free testosterone levels (18) were considered to have AD. To identify the origin of AD, the levels of LH and FSH were also measured. None of the patients in this group showed low levels of either FSH or LH. Thus, the AD of those patients can be considered caused by primary hypogonadism [26]. Only one male patient presented high LH levels, and one female patient showed high FSH levels.

The results of the comparison between patients with and without AD are included in Table 2. Different sex distribution was found between patients with and without AD (male:female, 8:9 vs. 13:5), but this difference did not reach significance (*p* = 0.176) (Table 2).

**Table 2:** Comparison between patients with or without androgen deficiency

	Androgenic deficiency		*p*-value
	No (*n* = 17)	Yes (*n* = 18)	
Age (years)	62.71 (SD = 11.58)	65.39 (SD = 8.68)	0.525^1^
Gender (male:female)	8:9	13:5	0.176^2^
Karnofsky < 70	1 (5.9%)	2 (11.1%)	1.000^2^
Resection			
Biopsy/partial	6 (35.3%)	4 (22.2%)	0.692^2^
Subtotal	3 (17.6%)	4 (22.2%)	
Total	8 (47.1%)	10 (55.6%)	
Ki67	26.29 (SD = 13.16)	29.33 (SD = 21.31)	0.970^1^
MGMT methylation	8 (50.0%)	9 (50.0%)	1.000^2^
Pattern of contrast enhancement			
Peripheric	8 (47.1%)	12 (66.7%)	0.315^2^
Heterogeneous	9 (52.9%)	6 (33.3%)	
Enhancing tumor (cc)	22.36 (SD = 19.11)	23.53 (SD = 11.58)	0.465^1^
Edema (cc)	59.78 (SD = 37.34)	51.20 (SD = 33.11)	0.533^1^
Necrosis (cc)	9.99 (SD = 11.33)	9.79 (SD = 10.62)	0.986^1^
Necrosis-to-contrast ratio	0.57 (SD = 0.74)	0.36 (SD = 0.28)	0.736^1^
AR expression (WB) (Z-value)	0.11 (SD = 1.07)	-0.12 (SD = 1.10)	0.818^1^
AR-Score (Z-value)	-1.15 (SD = 4.12)	0.11 (SD = 1.38)	0.251^1^
ARn/t fluorescence	0.16 (SD = 0.04)	1.28 (SD = 3.48)	0.573^1^
Progression-free survival (days)	135.0 [90.5–179.5]	252.0 [0–720.8]	0.041^3^
Men	200.0 [94.6–305.4]	252.0 [0–735.8]	0.155^3^
Women	105.0 [88.8–121.2]	286.0^a^ [32.2–540.8]	0.442^3^
Overall survival (days)	314.0 (95.2–532.8]	864.0 [333.9–1394.1]	0.156^3^
Men	314 [293.2–334.8]	406.0 [256.8–555.2]	0.257^3^
Women^b^	–	–	–

^1^Mann–Whitney *U* test

^2^Fisher’s exact test or Chi-square

^3^Log-rank test

^a^This value represents the mean period, whereas the mean could not be computed because of the limited follow-up

^b^No statistics were computed because all cases were censored

**Fig. 4:**
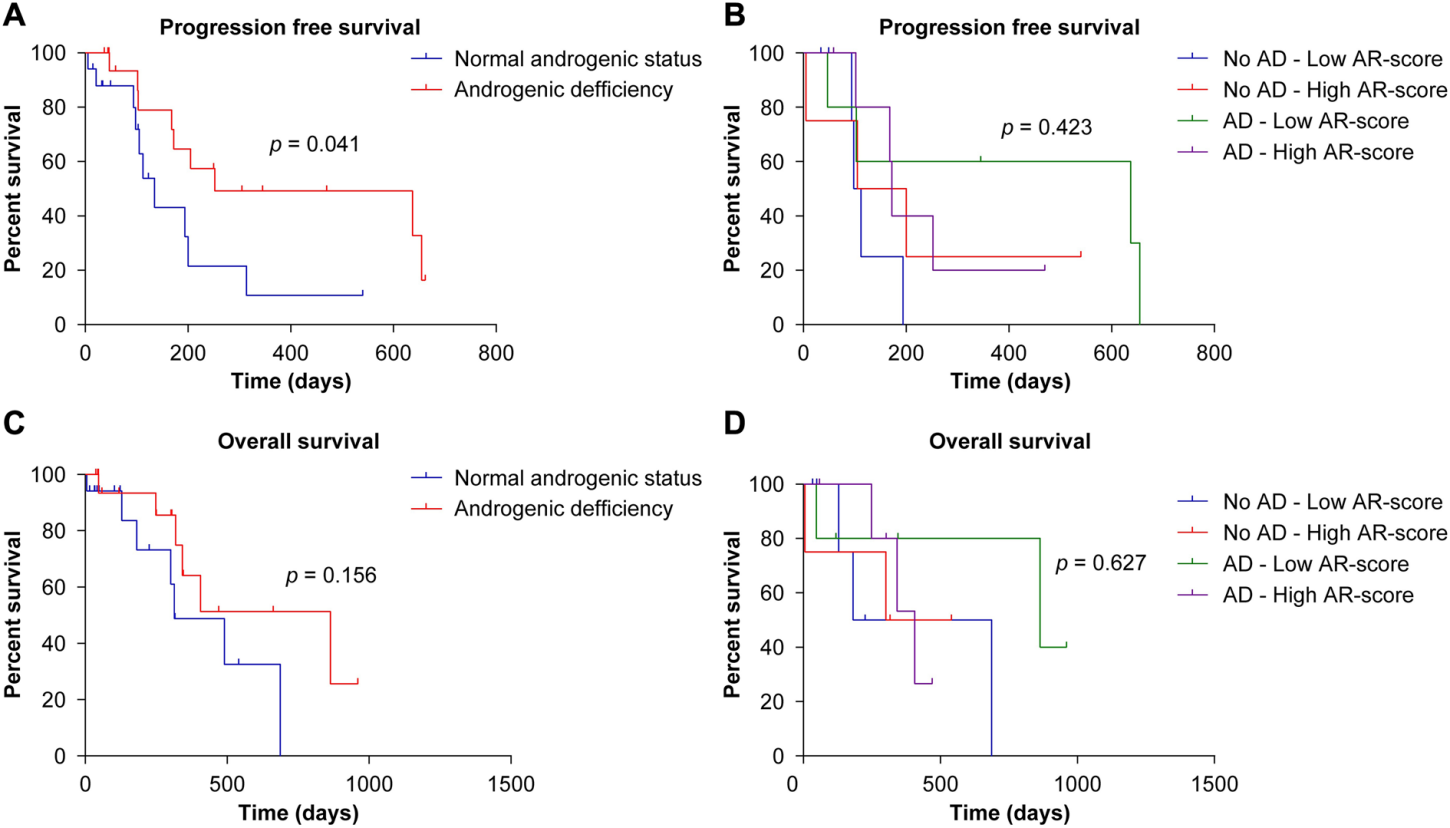
Survival analysis comparing the effect of androgen deficiency (AD). **A** Progression-free survival (PFS) analysis. **B** PFS analysis in patients with and without AD and different levels of AR scores. **C** Overall survival (OS) analysis. **D** OS analysis in patients with and without AD and different levels of AR scores

Interestingly, the PFS rate was higher in the AD group than in the non-AD group (252.0 vs. 135.0 days; *p* = 0.041) (Table 2, Fig. 4A, B). In a univariate regression analysis, patients with normal free testosterone levels had a significantly increased risk of progression (HR 2.704; 95% CI [1.007–7.257]; *p* = 0.048). In a multivariate regression model (including other variables that have been demonstrated to be associated with a worse prognosis in glioblastoma [i.e., age, extent of resection, presurgical KPS, and MGMT methylation status]), normal levels of circulating androgens upon diagnosis significantly increase the risk of progression (HR 6.346; 95% CI [1.812–22.223]; *p* = 0.004) (Table 3). On the contrary, regarding the OS, patients with AD presented a better prognosis than patients with normal androgenic status (864.0 vs. 314.0 days), but this difference was not significant (*p* = 0.156) (Table 2; Fig. 4C, D). In the univariate Cox regression analysis, a normal androgenic status was associated with a worse prognosis (HR 2.26; 95% CI [0.71–7–21]; *p* = 0.167). Although it did not reach significance in the univariate analysis, this variable was included in the model for multivariate analysis, and it significantly increased the risk for OS in patients with normal androgenic status (HR 5.18; 95% CI [1.20–22.46]; *p* = 0.028) (Table 3).

**Table 3:** Multivariate analysis for progression-free survival and overall survival, including the androgenic status in a model with the widely accepted prognostic factors in glioblastoma

Variable	Hazard ratio	95% confidence interval		*p*-value
		Lower	Upper	
Progression-free survival				
Age	1.070	1.01	1.13	0.016
Karnofsky < 70	0.000	0.00	-	0.983
Resection: s*ubtotal vs. biopsy*	1.043	0.22	5.05	0.958
Resection: c*omplete vs. biopsy*	0.617	0.18	2.08	0.435
MGMT methylation	2.239	0.78	6.44	0.135
Normal androgenic status	6.346	1.81	22.22	0.004
Overall survival				
Age	1.051	0.98	1.12	0.137
Karnofsky < 70	0.000	0.00	−	0.987
Resection: s*ubtotal vs. biopsy*	0.288	0.03	2.38	0.248
Resection: c*omplete vs. biopsy*	0.256	0.05	1.23	0.090
MGMT methylation	2.008	0.56	7.15	0.282
Normal androgenic status	5.188	1.20	22.46	0.028

Finally, an analysis of the prognosis in patients with or without AD regarding their AR score was also performed. Six patients in the AD group had high AR scores, whereas the other six patients presented a normal androgen status. The low number of patients in each group hinders achieving sufficient statistical power, but some interesting findings were obtained (Supplementary Table 1). As shown in Fig. 4B for PFS and 4D for OS, a tendency for better prognosis was observed in patients with both AD and low AR scores. In other words, AD appears to lose its “protective” effect when the AR activity is high.

## Discussion

This study focused on analyzing the relationship between circulating androgen levels and AR activity with the clinical, molecular, and radiological features of glioblastoma. The AR was widely expressed in the study patients, with a variable activity that was associated with a worse prognosis only in men. Finally, patients with AD presented a better prognosis than those with normal androgenic status.

All the study patients with available tissues show a significant AR expression. This result is in line with previous reports, where AR is commonly expressed in glioblastoma probes of both men and women [16]. However, the most important is not AR expression, but AR activity [23]. In this study, the AR activity has been inferred by the AR score, an index computed with the expression of specific ARGs. This index has been associated with prognosis in terms of OS in patients with glioblastoma [23]. In this study, the deleterious effect of the AR score was found only in men (Fig. 3C, D), although only 25 patients were included in this analysis. Furthermore, the extent of the resection was the only factor showing a significant difference between patients with high or low AR activity (Table 1). The extent of the resection is a well-known prognostic factor in glioblastoma [32]. Thus, this different distribution between patients with high and low AR activity may limit the value of the results presented here because patients with low AR scores presented account for higher proportion of patients with total resection (92.3%) than patients with high AR scores (41.7%). Regardless, it seems evident that the extent of resection does not appear to be a variable related to the AR-Score. It is associated with the tumor's location and its relationship with eloquent areas, but not with the molecular characteristics of the lesion. In any case, increasing the number of patients and measuring their AR activity will confirm whether the AR score is a good prognostic factor for glioblastoma. Furthermore, it is interesting to note the possible relationship between a higher proliferative index (Ki67) with increased androgen receptor activity, as well as a lower necrosis-to-contrast ratio. Although these results do not reach statistical significance, they support the existence of greater aggressiveness in glioblastomas with a high AR-Score.

AR activation is normally mediated by the union of an androgenic hormone (testosterone) to the receptor (so-called canonical pathway). Accordingly, circulating androgen levels were measured, and the androgenic status was evaluated. In this study, the presence of AD is an independent factor associated with a better prognosis in terms of PFS and OS. This finding is supported by previous works that have shown an increase in the growth and invasiveness of glioblastoma cell lines exposed to testosterone [17, 19, 20]. Furthermore, AR activation by its canonical pathway has been associated with the inhibition of the transforming growth factor-β pathway-induced antiproliferative and proapoptotic response in glioblastoma cells [12]. Higher levels of circulating androgens in patients with gliomas than in patients with other neurosurgical diseases were previously described [18], but no previous study has focused on the effect of circulating androgen levels on the prognosis of glioblastoma. The levels of testosterone have also be associated with a worse prognosis in prostate [33] and breast cancer [34]. Furthermore, the epidemiology of glioblastoma, with a higher incidence in men than in women (3:2) [2], also supports this finding. Unquestionably, at any moment of the disease course, men have higher possibility than women to have increased circulating androgen levels, which would explain the worse prognosis that was reported for men. Therefore, the higher the androgenic status is the environment in glioblastoma, the worse its behavior. Taken together our study and other pieces of evidence support the implication of the AR in glioblastoma pathogenesis mainly by its canonical pathway (i.e., androgen dependent). However, another interesting finding of the present study is the lack of a relationship between AR activity and circulating androgens levels. Interestingly, patients with AD were found to have high AR scores. This finding may have different explanations. Although the main activation pathway of the AR is the canonical pathway, there is an alternative pathway where the AR can be activated without hormone action (non-genomic pathway). Thus, a high AR activity in AD may be associated with the hormone-independent AR activation pathway. The hormone-independent AR pathway has mainly been studied in prostate and breast cancers [35–37] but much less in glioblastoma. An in vitro study with glioblastoma cell lines reported AR activation that was mediated by EGFR signaling [11]. In that study, the experiments were conducted without testosterone in the culture media. In this sense, and according to the previous paragraph, the presence of androgens would lead to preferential activation of the AR by its canonical pathway and not by the non-genomic one.

On the contrary, the presence of intratumoral steroidogenesis would also be a plausible explanation of the high AR activity in AD. As Lin et al. (2019) described, glioblastoma cells express all the machinery for steroid production, and there is an active intratumoral androgenic production [38]. Intratumoral steroidogenesis might not be regulated by circulating levels of androgens, as was described in prostate cancer [39], and it may significantly vary among patients, where some patients show higher steroid production, whereas others had lower production. The involvement of intratumoral steroidogenesis in glioblastoma growth, invasiveness, and recurrence should be deeply investigated.

Finally, another explanation for the higher AR activity in patients with AD is the cross-talk between steroid receptors. In this regard, cross-talk between glucocorticoid and androgenic pathways has been described. In many cases, steroid receptors have heterodimerization (e.g., glucocorticoid receptor [GR] and AR). Considering that dexamethasone is commonly used in patients with glioblastoma to control symptoms related to brain edema, the activation of the AR pathway via heterodimerization with GR may lead to an increase in the expression of ARGs and, consequently, an increase in the AR score. Dexamethasone has been associated with a worse prognosis in patients with glioblastoma, which is related with the direct action of this drug in glioblastoma cells [40]. The measurements of circulating androgen levels were performed before the initiation of dexamethasone treatment; thus, the levels of circulating androgens are not influenced by the drugs, but it would be the AR score.

Some limitations, apart from the low number of patients, should be considered. First, the AR activity has been inferred by the expression of a set of ARGs that has been validated by prostate cancer, but not for glioblastoma. A study demonstrated that the expressions of these ARGs are associated with a worse prognosis in glioblastoma, but a validation or identification of specific glioblastoma ARGs should be performed in future studies. On the contrary, the androgenic status is only validated before treatment initiation. Although no patient received supplementations for AD, this status may change along the disease. Future studies should consider the evolution of circulating androgen levels along the disease and confirm if persistent AD benefits patients with glioblastoma. To our knowledge, this is the first study reporting a relationship between the androgenic status and prognosis of patients with glioblastoma. This finding might have significant clinical and therapeutic implications for the management of this tumor.

## Conclusion

The presence of low circulating testosterone levels is associated with a better prognosis in glioblastoma in terms of both PFS and OS. This finding is in line with in vitro and in vivo studies where the exposure of glioblastoma cells to testosterone increases tumor aggressiveness. Nevertheless, AR activity may not be only mediated by the levels of circulating androgens. Alternative pathways may lead to AR activation, and they should be explored in future studies. In any case, the AR appears to be a good therapeutic target candidate for future clinical studies.

## Data Availability

The dataset generated during the current study is available from the corresponding author on reasonable request.

## References

[1] Ostrom QT, Gittleman H, Kruchko C, Barnholtz-Sloan JS (2019). Primary brain and other central nervous system tumors in Appalachia: regional differences in incidence, mortality, and survival. J Neurooncol.

[2] Lapointe S, Perry A, Butowski NA (2018). Primary brain tumours in adults. Lancet.

[3] Grochans S, Cybulska AM, Simińska D, Korbecki J, Kojder K, Chlubek D (2022). Epidemiology of glioblastoma multiforme-literature review. Cancers (Basel)..

[4] Chen B, Chen C, Zhang Y, Xu J (2021). Recent incidence trend of elderly patients with glioblastoma in the United States, 2000–2017. BMC Cancer.

[5] Gately L, McLachlan SA, Dowling A, Philip J (2017). Life beyond a diagnosis of glioblastoma: a systematic review of the literature. J Cancer Surviv.

[6] Bello-Alvarez C, Camacho-Arroyo I (2021). Impact of sex in the prevalence and progression of glioblastomas: the role of gonadal steroid hormones. Biol Sex Differ.

[7] Batistatou A, Kyzas PA, Goussia A, Arkoumani E, Voulgaris S, Polyzoidis K (2006). Estrogen receptor beta (ERβ) protein expression correlates with BAG-1 and prognosis in brain glial tumours. J Neurooncol.

[8] Hönikl LS, Lämmer F, Gempt J, Meyer B, Schlegel J, Delbridge C (2020). High expression of estrogen receptor alpha and aromatase in glial tumor cells is associated with gender-independent survival benefits in glioblastoma patients. J Neurooncol.

[9] Khalid H, Shibata S, Kishikawa M, Yasunaga A, Iseki M, Hiura T (1997). Immunohistochemical analysis of progesterone receptor and Ki-67 labeling index in astrocytic tumors. Cancer.

[10] González-Agüero G, Ondarza R, Gamboa-Domínguez A, Cerbón MA, Camacho-Arroyo I (2001). Progesterone receptor isoforms expression pattern in human astrocytomas. Brain Res Bull.

[11] Zalcman N, Gutreiman M, Shahar T, Weller M, Lavon I (2021). Androgen receptor activation in glioblastoma can be achieved by ligand-independent signaling through EGFR-A potential therapeutic target. Int J Mol Sci.

[12] Yu X, Jiang Y, Wei W, Cong P, Ding Y, Xiang L (2015). Androgen receptor signaling regulates growth of glioblastoma multiforme in men. Tumour Biol.

[13] Culig Z, Santer FR (2014). Androgen receptor signaling in prostate cancer. Cancer Metastasis Rev.

[14] Heemers HV, Tindall DJ (2007). Androgen receptor (AR) coregulators: a diversity of functions converging on and regulating the AR transcriptional complex. Endocr Rev.

[15] Zalcman N, Canello T, Ovadia H, Charbit H, Zelikovitch B, Mordechai A (2018). Androgen receptor: a potential therapeutic target for glioblastoma. Oncotarget.

[16] Zhao N, Wang F, Ahmed S, Liu K, Zhang C, Cathcart SJ (2021). Androgen receptor, although not a specific marker for, is a novel target to suppress glioma stem cells as a therapeutic strategy for glioblastoma. Front Oncol.

[17] Daswani B, Khan Y (2021). Insights into the role of estrogens and androgens in glial tumorigenesis. J Carcinog.

[18] Bao D, Cheng C, Lan X, Xing R, Chen Z, Zhao H (2017). Regulation of p53wt glioma cell proliferation by androgen receptor-mediated inhibition of small VCP/p97-interacting protein expression. Oncotarget.

[19] Rodríguez-Lozano DC, Piña-Medina AG, Hansberg-Pastor V, Bello-Alvarez C, Camacho-Arroyo I (2019). Testosterone promotes glioblastoma cell proliferation, migration, and invasion through androgen receptor activation. Front Endocrinol (Lausanne).

[20] Orozco M, Valdez RA, Ramos L, Cabeza M, Segovia J, Romano MC (2020). Dutasteride combined with androgen receptor antagonists inhibit glioblastoma U87 cell metabolism, proliferation, and invasion capacity: androgen regulation. Steroids.

[21] Kim HJ, Kim T-J, Kim YG, Seong C, Cho J-H, Kim W (2021). Antioxidant and antiproliferative activity of finasteride against glioblastoma cells. Pharmaceutics.

[22] Werner CK, Nna UJ, Sun H, Wilder-Romans K, Dresser J, Kothari AU (2020). Expression of the androgen receptor governs radiation resistance in a subset of glioblastomas vulnerable to antiandrogen therapy. Mol Cancer Ther.

[23] Fariña-Jerónimo H, de Vera A, Medina L, Plata-Bello J (2022). Androgen receptor activity is associated with worse survival in glioblastoma. J Integr Neurosci.

[24] Morris PD, Malkin CJ, Channer KS, Jones TH (2004). A mathematical comparison of techniques to predict biologically available testosterone in a cohort of 1072 men. Eur J Endocrinol.

[25] Bhasin S, Brito JP, Cunningham GR, Hayes FJ, Hodis HN, Matsumoto AM (2018). Testosterone therapy in men with hypogonadism: an endocrine society clinical practice guideline. J Clin Endocrinol Metab.

[26] Morales A, Bebb RA, Manjoo P, Assimakopoulos P, Axler J, Collier C (2015). Diagnosis and management of testosterone deficiency syndrome in men: clinical practice guideline. CMAJ.

[27] Schmittgen TD, Livak KJ (2008). Analyzing real-time PCR data by the comparative CT method. Nat Protoc.

[28] Nelson PS, Clegg N, Arnold H, Ferguson C, Bonham M, White J (2002). The program of androgen-responsive genes in neoplastic prostate epithelium. Proc Natl Acad Sci U S A.

[29] Abeshouse A, Ahn J, Akbani R, Ally A, Amin S, Andry CD (2015). The molecular taxonomy of primary prostate cancer. Cell.

[30] Acosta-Lopez S, Diaz-Bethencourt D, Concepción-Massip T, Martin-Fernandez de Basoa MC, Plata-Bello A, Gonzalez-Rodriguez A, et al. The androgen receptor expression and its activity have different relationships with prognosis in hepatocellular carcinoma. Sci Rep. 2020;10:22046. 10.1038/s41598-020-79177-2 PMC7744520 33328560

[31] Juan-Albarracín J, Fuster-Garcia E, García-Ferrando GA, García-Gómez JM (2019). ONCOhabitats: A system for glioblastoma heterogeneity assessment through MRI. Int J Med Inform.

[32] Brown TJ, Brennan MC, Li M, Church EW, Brandmeir NJ, Rakszawski KL (2016). Association of the extent of resection with survival in glioblastoma: a systematic review and meta-analysis. JAMA Oncol.

[33] Shaneyfelt T, Husein R, Bubley G, Mantzoros CS (2000). Hormonal predictors of prostate cancer: a meta-analysis. J Clin Oncol.

[34] Farhat GN, Cummings SR, Chlebowski RT, Parimi N, Cauley JA, Rohan TE (2011). Sex hormone levels and risks of estrogen receptor-negative and estrogen receptor-positive breast cancers. JNCI Journal of the National Cancer Institute.

[35] Leung JK, Sadar MD. Non-Genomic Actions of the Androgen Receptor in Prostate Cancer. Front Endocrinol (Lausanne). 2017;8. 10.3389/fendo.2017.00002 PMC5239799 28144231

[36] Kono M, Fujii T, Lim B, Karuturi MS, Tripathy D, Ueno NT (2017). Androgen Receptor Function and Androgen Receptor-Targeted Therapies in Breast Cancer: A Review. JAMA Oncol.

[37] Tindall D, Lonergan P (2011). Androgen receptor signaling in prostate cancer development and progression. J Carcinog.

[38] Lin H-Y, Ko C-Y, Kao T-J, Yang W-B, Tsai Y-T, Chuang J-Y (2019). CYP17A1 maintains the survival of glioblastomas by regulating sar1-mediated endoplasmic reticulum health and redox homeostasis. Cancers (Basel)..

[39] Armandari I, Hamid AR, Verhaegh G, Schalken J (2014). Intratumoral steroidogenesis in castration-resistant prostate cancer: a target for therapy. Prostate Int.

[40] Aldaz P, Auzmendi-Iriarte J, Durántez M, Lasheras-Otero I, Carrasco-Garcia E, Zelaya MV (2021). Identification of a dexamethasone mediated radioprotection mechanism reveals new therapeutic vulnerabilities in glioblastoma. Cancers (Basel)..

